# Mechanical Performance and Microstructural Characterization of Light Alloys

**DOI:** 10.3390/ma16175915

**Published:** 2023-08-29

**Authors:** Qinghuan Huo

**Affiliations:** School of Materials Science and Engineering, Central South University, Changsha 410083, China; huoqinghuan@csu.edu.cn

The present Special Issue titled “Mechanical Performance and Microstructural Characterization of Light Alloys” aims to report the close relation between mechanical performance and microstructure in light alloys, such as Al, Mg, Ti, and their alloys. Moreover, it concerns any improvement in mechanical properties or any optimization in microstructural characterization.

Strong interactions between precipitates and dislocations are the common strengthening route for Al alloys. However, the local stress concentration will be induced along with the strengthening [1,2,3]. Under this context, one confused question has come out: what is the balance between continuous strengthening and accumulated damage? This requires a comprehensive study on microstructural development, damage analysis, and combinations with calculations. Similar difficulties are also found in Mg alloys, which is the lightest structural material. Classical experience that discontinuous precipitation would deteriorate the mechanical performance may break down under certain service environments [4]. Detailed reasons are still being researched for and characterized. More recently, additive manufacturing and dissimilar metal welding have attracted much attention for its various advantages, such as high efficiency and easy repeatability. Nonetheless, due to the manufacturing characteristics [5,6,7], mechanical anisotropy and performance distribution may become inhomogeneous and further deep microstructural characterizations should be performed.

To further trigger the development of light alloys, the present Special Issue has not only collected excellent studies on their mechanical performance and microstructural characterization but has also welcomed studies on any optimized processing methods, advanced characterization techniques, and novel prospects on manufacturing for light alloys.

## Data Availability

Not applicable.
